# Docetaxel suppressed cell proliferation through Smad3/HIF-1α-mediated glycolysis in prostate cancer cells

**DOI:** 10.1186/s12964-022-00950-z

**Published:** 2022-12-19

**Authors:** Junming Peng, Zhijun He, Yeqing Yuan, Jing Xie, Yu Zhou, Baochun Guo, Jinan Guo

**Affiliations:** 1 The Department of Urology, Shenzhen People’s Hospital (The First Affiliated Hospital, Southern University of Science and Technology; The Second Clinical Medical College, Jinan University), Shenzhen Urology Minimally Invasive Engineering Center, Shenzhen, 518055 Guangdong China; 2 Department of Pharmacy, Zhuhai Center for Maternal and Child Health Care, Zhuhai, 519000 China; 3 Shenzhen Public Service Platform on Tumor Precision Medicine and Molecular Diagnosis, Shenzhen, China; 4 Shenzhen Key Laboratory of Kidney Diseases (ZDSYS201504301616234), Shenzhen People’s Hospital (The Second Clinical Medical College, Jinan University; The First Affiliated Hospital, Southern University of Science and Technology), Shenzhen, 518055 Guangdong China; 5 Department of Nephrology, Shenzhen People’s Hospital (The Second Clinical Medical College, Jinan University; The First Affiliated Hospital, Southern University of Science and Technology), Shenzhen, 518020 Guangdong China; 6 Department of Urology, Shenzhen People’s Hospital, The Second Clinical College of Jinan University, Shenzhen, 518000 China

**Keywords:** Docetaxel, Tumor glycolysis, Smad3, Cell proliferation, Prostate cancer

## Abstract

**Background:**

Tumor glycolysis is a critical event for tumor progression. Docetaxel is widely used as a first-line drug for chemotherapy and shown to have a survival advantage. However, the role of docetaxel in tumor glycolysis remained poorly understood.

**Methods:**

The effect of Docetaxel in tumor glycolysis and proliferation were performed by CCK-8, Western blotting, real-time PCR, glucose, and lactate detection and IHC. ChIP and luciferase assay were used to analyze the mechanism of Docetaxel on Smad3-mediated HIF-1α transactivity.

**Results:**

In this study, we showed that docetaxel treatment led to a significant inhibition of cell proliferation in prostate cancer cells through PFKP-mediated glycolysis. Addition of lactate, a production of glycolysis, could reverse the inhibitory effect of docetaxel on cell proliferation. Further analysis has demonstrated that phosphorylation of Smad3 (Ser213) was drastically decreased in response to docetaxel stimulation, leading to reduce Smad3 nuclear translocation. Luciferase and Chromatin immunoprecipitation (ChIP) analysis revealed that docetaxel treatment inhibited the binding of Smad3 to the promoter of the *HIF-1α* gene, suppressing transcriptional activation of HIF-1α. Moreover, ectopic expression of Smad3 in prostate cancer cells could overcome the decreased HIF-1α expression and its target gene PFKP caused by docetaxel treatment. Most importantly, endogenous Smad3 regulated and interacted with HIF-1α, and this interaction was destroyed in response to docetaxel treatment. What’s more, both HIF-1α and PFKP expression were significantly reduced in prostate cancer received docetaxel treatment in vivo.

**Conclusion:**

These findings extended the essential role of docetaxel and revealed that docetaxel inhibited cell proliferation by targeting Smad3/HIF-1α signaling-mediated tumor Warburg in prostate cancer cells.

**Video Abstract**

**Supplementary Information:**

The online version contains supplementary material available at 10.1186/s12964-022-00950-z.

## Background

Dysfunction of glycolysis facilitated many advantages of tumor cells, which has been characterized as a hallmark of cancer [1, 2]. Tumor glycolysis, also named Warburg effect, has been demonstrated to play an critical role in development of cancer, such as tumor growth [3], immune evasion [4], metastasis [5], recurrence [6], sensitivity [7] and angiogenesis [8]. In addition to tumor glycolysis, metabolic reprogramming, including De novo* lipogenesis* (DNL) and polyol pathway, has linked the interaction between tumor cell and other component cell in tumor microenvironment to tumor progression, such as immune cells, inflammatory cells, stomal cells [2]. The evidence suggested that the Warburg effect is becoming a promising target for diagnosis and therapy. A further better understanding of the tumor glycolysis underlying tumor biological process is essential to develop effective therapeutic.

Tumor glycolysis, a characteristic tumor cell phenotype, can expedite tumor progression by enhancement of lactate production and glucose uptake. The dysregulation of glycolysis not only provided tumor cells with nutrients and ATP to meet tumor proliferation and growth, but also remodeled an acidic environment that results in demolition of extracellular matrix and expedites growth and metastasis [9, 10], which was attributed to increased critical glycolytic enzymes expression, including glucose transporter isoform 1 (GLUT1), hexokinase 2 (HK2), Phosphofructokinase, platelet (PFKP), Pyruvate kinase isozymes (PKM), Lactate dehydrogenase A (LDHA), in a serial of human tumors. What’s more, hypoxia-inducible factor-1α (HIF-1α) is a key transcriptional regulatory protein that regulates an arrangement of hypoxia-sensitive proteins expression, such as PKM2, HK2, PFKP, to preserve the survival of tissue in tumor microenvironment. What’s more, Smad3 also has been reported to play a critical role in metabolic reprogramme in tumor progression, including tumor glycolysis [11], glutaminolysis [12], lipid metabolism [13]. Interestingly, the work from Xu et al. study showed that endogenous Smad3 regulated and interacted with HIF-1α in colorectal cancer cells. These findings implied the crosstalk between Smad3 and HIF-1α played a critical role in tumor progression.

Docetaxel, the first-line chemotherapeutic agent for advanced stage prostate cancer [14], is gradually gained widespread attention due to its novel function has been identified [15, 16], However, the toxic side effects and drug resistance limited clinical success of Docetaxel. Early staged and localized PCa can be well controlled by prostatectomy or radiotherapy. For locally advanced and metastatic PCa, androgen deprivation therapy (ADT) is currently considered as the most effective treatment, giving a 70% initial effective rate. The most studied factor responsible for these effects is hypoxia-inducible factor-1 (HIF-1) that significantly contributes to the aggressiveness and chemoresistance of different tumors [17]. In addition to HIF-1, Smad3 also has been reported to induce chemoresistance in various of cancer [18, 19]. In this study, we further showed that docetaxel treatment significantly inhibited cell proliferation in prostate cancer cells by inhibition of Smad3-mediated tumor glycolysis, leading to decrease glucose uptake and lactate production, which illustrated a novel function of docetaxel in prostate cancer cells. Docetaxel stimulation led to a significant downregulated the expression of HIF-1α and PFKP, but not PKM2, resulting in suppression of lactate production, glucose uptake, and cell proliferation. These findings revealed that docetaxel-Smad3/HIF-1α axis regulated tumor glycolysis in prostate cancer cells, establishing the central role Samd3/HIF-1α in docetaxel-mediated tumor Warburg effect and tumor progression.

## Methods

### Chemical reagents and antibodies

Docetaxel (ID0400) and lactate (L8601) obtained from Solarbio life science (Beijing, China). Lactate assay kits (KA0833) was purchased from Abnova. The SimpleChIP® Enzymatic Chromatin IP Kit (Magnetic Beads) (9003) was from Cell Signaling Technology (Danvers, MA, USA). Glucose uptake fluorometric assay kit (MAK084) was purchased from Sigma Aldrich (St. Louis, MO, USA). Antibodies targeting PKM2 (Abcam, ab137852,1:2000 for WB), HIF-1α (Abcam, ab114977,1:2000 for WB), PFKP (Abcam, ab119796,1:2000 for WB), α-tubulin (Abcam, ab7291,1:4000 for WB) and β-actin (Abcam, ab8226,1:4000 for WB) were purchased from Abcam Company (MA, USA); Fetal boive serum (FBS), Alexa-488- and 594-conjugated secondary antibodies were from Invitrogen (Thermo Fisher Scientific). The trizol was purchased from Invitrogen and All-in-One First-Strand cDNA Synthesis Kit and All-in-One qPCR Mix were obtained from GeneCopoeia (Guangzhou, China). Subcellular fractionation was conducted using NE-PER™ Nuclear and Cytoplasmic Extraction Reagents (thermo fisher) following the manufacturer’s recommendations. All ultrapure reagents were from Promega (Madison, WI, USA).

### Cell culture and treatment

PC-3M, PC-3M IE8 cells and DU145 were purchased from American Type Culture Collection (ATCC, Manassas, VA) and cultured according to the manufacturer’s recommendations. For treatment, the prostate cancer cells were treated with Docetaxel at concertation of 5 uM or lactate (5 uM), respectively. While plasmids were transfected into cells with lipofectamine3000 (L‐3000) following the manufacturer's instructions.

### RNA extraction and real-time PCR

As described in Xu et al. study [20], the whole RNA was extracted with Trizol reagents according to the instruction. Reverse transcription and quantitative PCR (qPCR) were performed using the All-in-One First-Strand cDNA Synthesis Kit and All-in-One qPCR Mix (GeneCopoeia) according to the manufacturer’s protocol. The primers used in this study as followed: HIF-1α forward, 5′-GAACGTCGAAAAGAAAAGTCTCG-3’ and 5′-CCTTATCAAGATGCGAACTCACA-3′; PFKP forward, 5′-CGCCTACCTCAACGTGGTG-3′, and reverse, 5’- ACCTCCAGAACGAAGGTCCTC-3’; HK2 forward, 5’- TGCCACCAGACTAAACTAGACG-3’ and reverse, 5’- CCCGTGCCCACAATGAGAC-3’; β-actin forward, 5’-CGCGAGAAGATGCCCAGATC-3’ and reverse, 5’-TCACCGGAGTCCATCACGA-3’.

### Subcellular fractionation and western blotting

Subcellular fractionation was performed using Subcellular fractionation was conducted using NE-PER™ Nuclear and Cytoplasmic Extraction Reagents according the manufacturer’s recommendations. Western blotting was performed. Briefly, the protein was separated by SDS-PAGE and transferred into nitrocellulose transfer membrane to further incubation with 5% (w/v) milk in PBS/0.05% (v/v) Tween‐20 for 1 h, the incubation for the membrane with the indicated antibody overnight at 4 °C, subsequently followed by incubation with a horseradish peroxidase secondary antibody for another 1 h at room temperature. Proteins were showed using an enhanced chemiluminescence (Perkin Elmer).

### Lactate production and glucose composition

PC-3M and PC-3M IE8 cells were stimulated with or without docetaxel for 48 h. The condition medium was directly assayed using the lactate and glucose detection Kit according to the manufacturer’s protocol, respectively.

### CCK-8 assay

The cell viability of PC-3M and PC-3M IE8 cells were determined by a Cell Counting Kit-8 (CCK-8) (Dojindo, Japan). Briefly, cells were reseeded into a 96-well plate at a density of 6 × 10^4^ cells/well followed by docetaxel stimulation for a time course. At post-treatment, the medium was replaced with 100ul medium contained 10 μl CCK-8 to incubate for 1 h at 37 °C, and absorbance were measured at 450 nm. The assays were performed in triplicate.

### Chromatin immunoprecipitation (ChIP) assay

ChIP was conducted as described in Zhang et al. study [21]. Briefly, ChIP was performed using an anti-Smad3 antibody or rabbit IgG (Millipore, Billerica, MA, USA) to pulldown chromatin extracts equivalent to 5 × 10^5^ cells. ChIP samples were quantified by qPCR (SYBR Green Master Mix; Applied Biosystems) and the ChIP qPCR data were normalized using the percent input method. The ChIP primer used in the study for HIF-1α: forward: GGTCACTTCCTCCCACCTAAT; Reverse: CAGGCTCACGCTACGGAATC.

### Luciferase assay

Cells were reseeded in 12-well plates at a density of 1 × 10^5^ cells /well, and co-transfected with pcDNA3.1 or pcDNA-Smad3 and plasmid containing HIF-1α promoter regions. At 24 h post-transfection, cells were treated with or without 5 uM docetaxel for another 48 h. The dual-luciferase reporter assay was performed using the Dual-luciferase Reporter Assay System (Promega). Renilla luciferase was used as the internal reference to verify the transfection efficiency and calculate the relative luciferase activity. i.e., the ratio of Firefly luciferase activity to renilla luciferase activity.

### Immunohistochemistry (IHC)

IHC was performed as described in their work [21, 22]. The deparaffinized and rehydrated sections were blocked, and then incubated with the indicated antibody at 4 °C overnight, the bound antibodies were then visualized using diaminobenzidine. The slides were also counterstained with hematoxylin. The positive staining area was taken at 400 × magnification on each slide and quantified using ImagePro Plus 6.0 software (Media Cybernetics, Rockville, MD, USA).

### Hematoxylin and eosin

Tissues were fixed in 4% paraformaldehyde for 24 h, dehydrated, embedded in paraffin, and sliced into 4-μm-thick sections. HE staining were performed using specific kits from Solarbio Life Sciences Co. Ltd. (Beijing, China) as per the manufacturer's instructions.

### In vivo mice experiments

The animal was performed according to animal ethical committee instruction. Briefly, 1 × 10^5^ PC-3M prostate cancer cells were injected into null mouse. Once tumors were palpable, mice were randomized into the following groups (5 mice per group): (A) control (vehicle; 5% dextrose); (B) 10 nM (dissolved in 5% dextrose) administered by intraperitoneal injection once daily. Tumor volumes were monitored for the next 2 weeks. After tumors were excised, tumor weight was measured and tumor volume was calculated according to the formula (W^2^ × L)/2, where W is width and L is length of the tumor.

### Statistical analysis

Data analysis was performed using the GraphPad Prism V software (La Jolla, CA, USA). A difference in comparison with a *p* less than 0.05 was considered statistically significant. Statistical differences among groups were determined by Student’s t-test, one-way analysis of variance (ANOVA) was used to determine the significance for mRNA and intensity quantified.

## Results

### Docetaxel suppressed cell proliferation and aerobic glycolysis

**Fig. 1:**
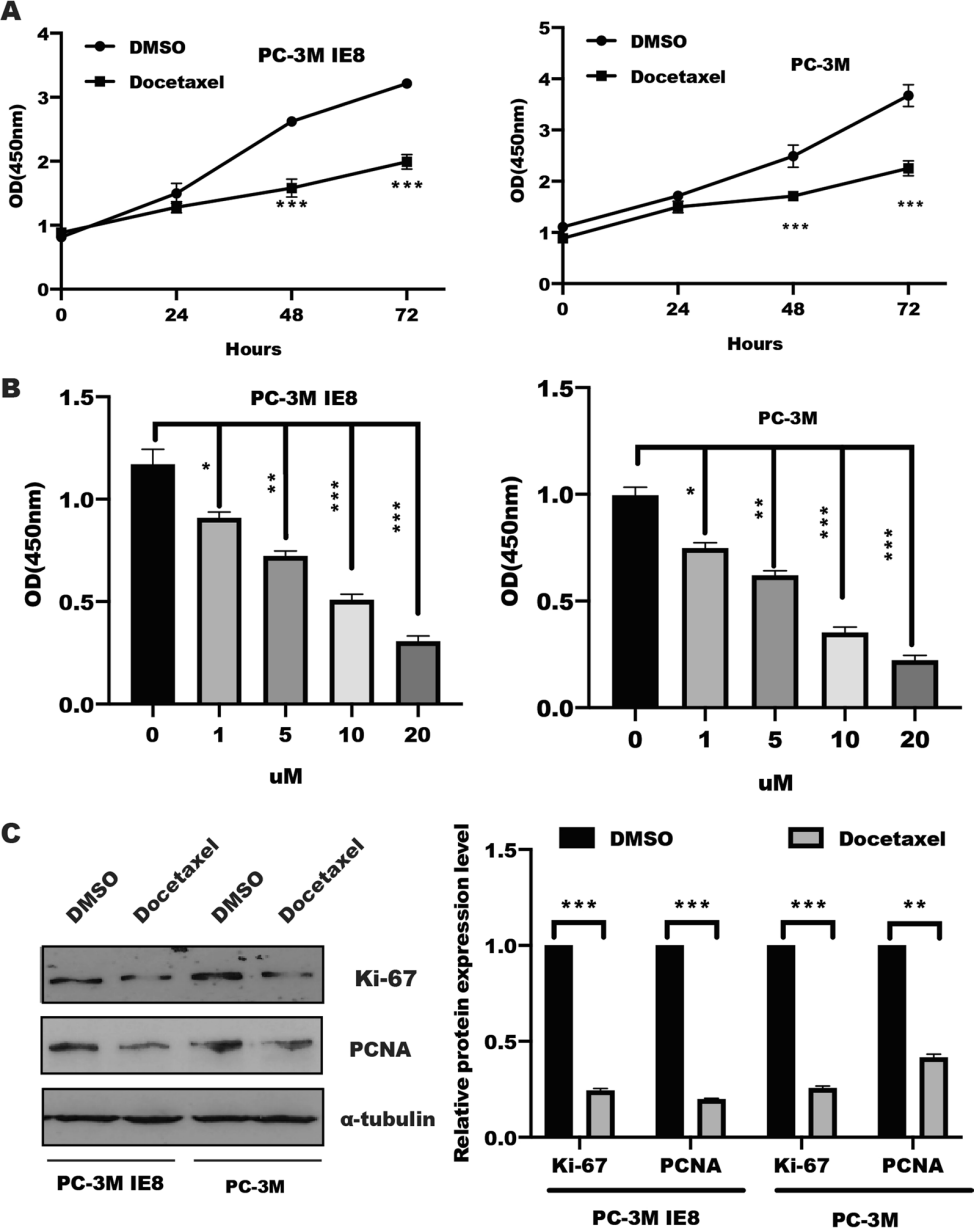
Docetaxel suppressed cell proliferation. **A** PC-3M and PC-3M IEC8 cells were digested and reseeded in a 96-well plate, respectively, and treated with docetaxel (5 uM) in a time course. The cell viability of PC-3M and PC-3M IE8 cells was determined by CCK-8 assay. Data represented the mean ± s.d. of three independent experiments and were analyzed by the one sample t test for significance versus the control group. ***P < 0.001. **B** After digestion, PC-3M IE8 and PC-3M cells were reseeded in a density of 10^5^ in 96-well plate overnight, the cells were treated with docetaxel in a various of concentration for 48 h. The cell viability was measured by CCK-8 assay. Data represented the mean ± s.d. of three independent experiments and were analyzed by the one sample t test for significance versus the control group. *P < 0.05, **P < 0.01, ***P < 0.001. **C** Western blotting was performed to analyze cell proliferation characterized by PCNA and Ki-67 expression in PC-3M IE8 and PC-3M cells treated with or without docetaxel (5 uM) for 48 h, α-tubulin served as internal control and the intensity of bands was quantified and analyzed by by t test for significance versus the control group, Data represented the mean ± s.d. of three independent experiments, **P < 0.01, ***P < 0.001

Docetaxel is a first-line chemotherapy for the treatment of patients with castration-resistant prostate cancer (CRPC) and has been demonstrated to be play a suppressive function in cell proliferation and apoptosis [23]. Consisted with this, our results showed that PC-3M, PC-3M IE8 and DU145 cells treated with docetaxel led to a significant inhibition of cell proliferation in a time- and dose-dependent manner (Fig. 1A–B, Additional file 1: Fig. S1). In line with the study from Niu et al. [24], 5 uM was choose in the whole study, the western blotting analysis also showed that both PCNA and Ki-67, a cell proliferation indicator, were drastically decreased in PC-3M and PC-3M IE8 cells in response to docetaxel stimulation (Fig. 1C–D).

### Docetaxel regulated tumor glycolysis in prostate cancer cells in HIF-1a dependent manner.

**Fig. 2:**
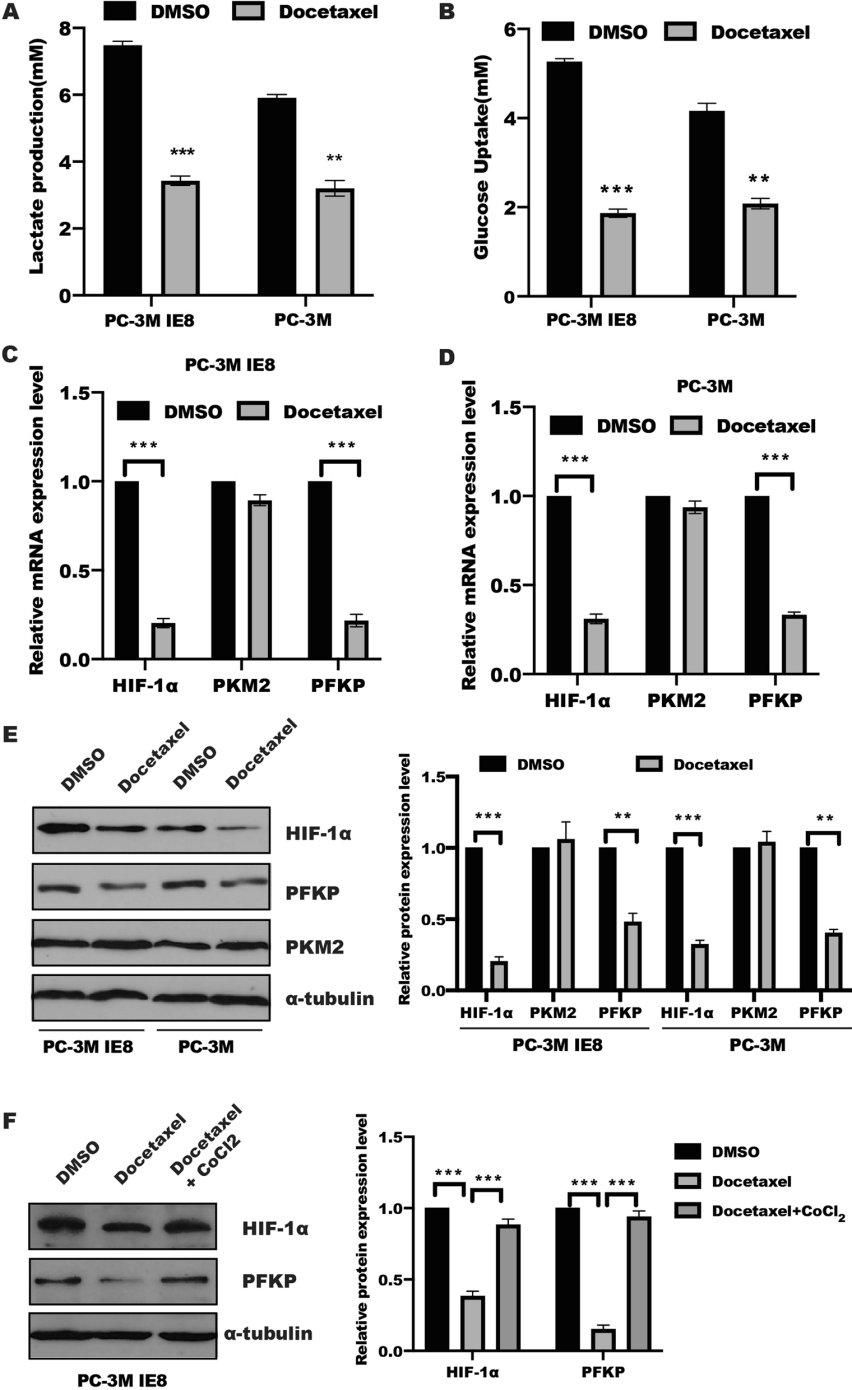
Docetaxel inhibited HIF-1α-mediated tumor glycolysis. **A**–**B** PC-3M IE8 or PC-3M cells were treated with or without docetaxel (5 uM) for 48 h, the level of lactate and glucose in the supernatant were measured according to the instruction. Data represented the mean ± s.d. of three independent experiments and were analyzed by t test for significance versus the control group. **P < 0.01, ***P < 0.001. The total RNA was extracted using trizol reagents, and Real-time PCR was performed to analyze the glycolytic genes expression in PC-3M IE8 (**C**) and PC-3M cells (**D**), Data represented the mean ± s.d. of three independent experiments and were analyzed by the one sample t test for significance versus the control group. ***P < 0.001. **E** Western blotting was employed to detect indicated proteins in PC-3M IE8 and PC-3M cells treated with or without docetaxel for 48 h, α-tubulin served as internal control and the bands intensity were quantified and analyzed by t test for significance versus the control group. Data represented the mean ± s.d. of three independent experiments, **P < 0.01, ***P < 0.001. **F** PC-3M IE8 cells were treated with docetaxel (5 uM) for 1 h, following by CoCl_2_ stimulation for further 48 h. The total protein was harvested and detected indicated proteins by SDS-PAGE. A quantification of each blot is indicated. α-tubulin served as internal control, data represented the mean ± s.d. of three independent experiments and analyzed by One-way ANOVA with multiple comparisons, followed by Bonferroni post hoc test for significance. ***P < 0.001

Reprogrammed glucose metabolism of enhanced Warburg effect (or aerobic glycolysis) is considered as a hallmark of cancer [25], which is important in tumor progression. To explore whether tumor glycolysis involved in docetaxel regulated cell proliferation. We found that docetaxel treatment (10 nM) led to a significant reduction of the level of lactate production and glucose consumption (Fig. 2A–B). Real-time PCR and western blotting were performed to further determine the effect of docetaxel on glycolytic gene expression in prostate cancer cells. As shown in Fig. 2C–D, compared to the control group, docetaxel stimulation in PC-3M and PC-3M IE8 cells led to a downregulation of glycolytic genes, such as HIF-1α and PFKP, no significance difference was observed in PKM2 expression. In line with this, the protein level of HIF-1α, HK2, and PFKP were decreased in PC-3M and PC-3M IE8 cells with docetaxel stimulation for 48 h (Fig. 2E), the similar results were obtained in DU145 cells (Additional file 1: Fig. S1). These findings suggested that docetaxel inhibited tumor glycolysis.

HIF-1α has been demonstrated to play an critical role in tumor angiogenesis [26, 27], cell proliferation and growth [3], and energy metabolism [28, 29]. To test whether docetaxel-mediated glycolysis in HIF-1α dependent manner. CoCl2-induced HIF-1α expression could reverse the inhibitory effect of docetaxel in tumor glycolysis, including glycolytic genes expression, lactate production and glucose uptake (Fig. 2F). These results imply that docetaxel regulated aerobic glycolysis through HIF-1α-mediated activation of glycolytic genes in prostate cancer cells.

### Docetaxel regulated glycolysis through Smad3

**Fig. 3:**
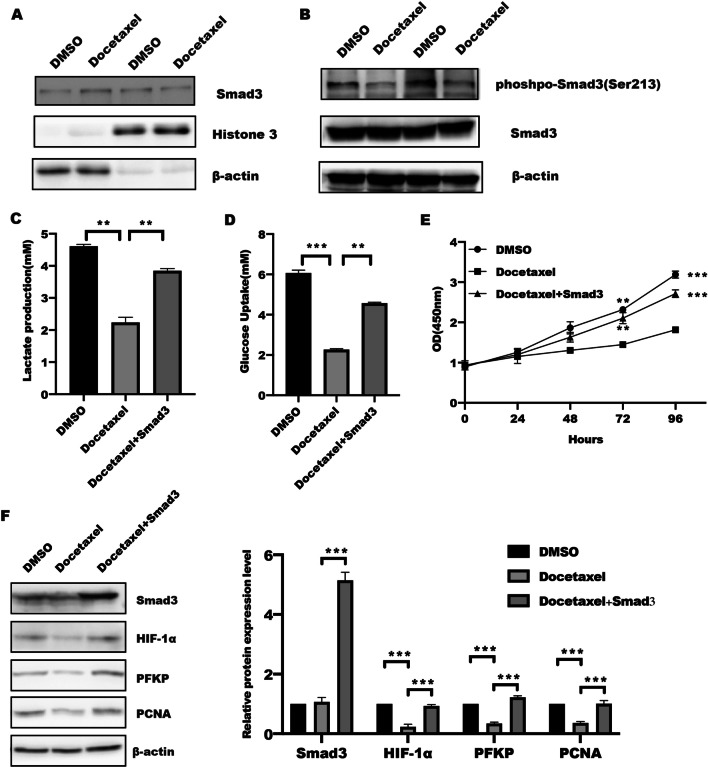
Docetaxel regulated tumor glycolysis and growth in Smad3-dependnet way. **A** PC-3M cells were serum starved for 24 h and then left untreated or treated with docetaxel for an additional 1 h. Levels of cytosolic and nuclear Smad3 were determined by western blotting. α-tubulin and histone 3 were used as internal controls for the cytosolic and nuclear fractions. **B** The total protein was collected from PC-3M cells treated with shikonin for 1 h and separated by SDS-PAGE to detect the phosphorylation level of Smad3 (Ser213). PC-3M cells were treated with or without docetaxel for 24 h, and following by transfection with or without Smad3 plasmid for further 48 h, the level of lactate production (**C**), glucose consumption (**D**), and cell viability (**E**) were measured according to instruction, **F** the total protein was collected and subject by SDS-PAGE to detect indicated proteins, the band intensity was quantified, data represented the mean ± s.d. of three independent experiments and analyzed by One-way ANOVA with multiple comparisons, followed by Bonferroni post hoc test for significance. **P < 0.01, ***P < 0.001

The pervious study has demonstrated that Smad3, a center mediator of TGFβ [11, 13, 30–32], is critical player in modulating energy metabolism. Phosphorylation of Smad3 linker domain (pSmad3L) indirectly inhibited its COOH-terminal phosphorylation, and the proliferative effect mediated by RTK-dependent pSmad3L pathway antagonized TGF-β signaling through the cytostatic pSmad3C pathway in normal epithelial cells [33]. Based on these, we tried to identify the effect of docetaxel in the phosphorylation of Smad3 linker. As shown in Fig. 3A, the immunoblotting results showed that Smad3 nuclear translocation was decreased in response to docetaxel stimulation for 1 h, which was attributed to the phosphorylation of Smad3 (Ser213) at the linker region was significantly reduced in PC-3M and PC-3M IE8 cells with docetaxel stimulation (Fig. 3B).

To further confirm Smad3 served a critical role in docetaxel-mediated glycolysis, we transfected plasmid Smad3 into PC-3M IE8 cells by transient transfection. We found that ectopic expression of Smad3 reversed the inhibitory effect of docetaxel on tumor glycolysis and cell proliferation (Fig. 3C–E). Further analysis showed that Smad3 overexpression in prostate cancer cells could overcome the decreased HIF-1α and PFKP caused by docetaxel (Fig. 3F). These findings implied that docetaxel treatment suppressed HIF-1α-mediated glycolysis and proliferation in Smad3-dependent manner.

### Smad3 is required for docetaxel-mediated HIF-1α transactivation

**Fig. 4:**
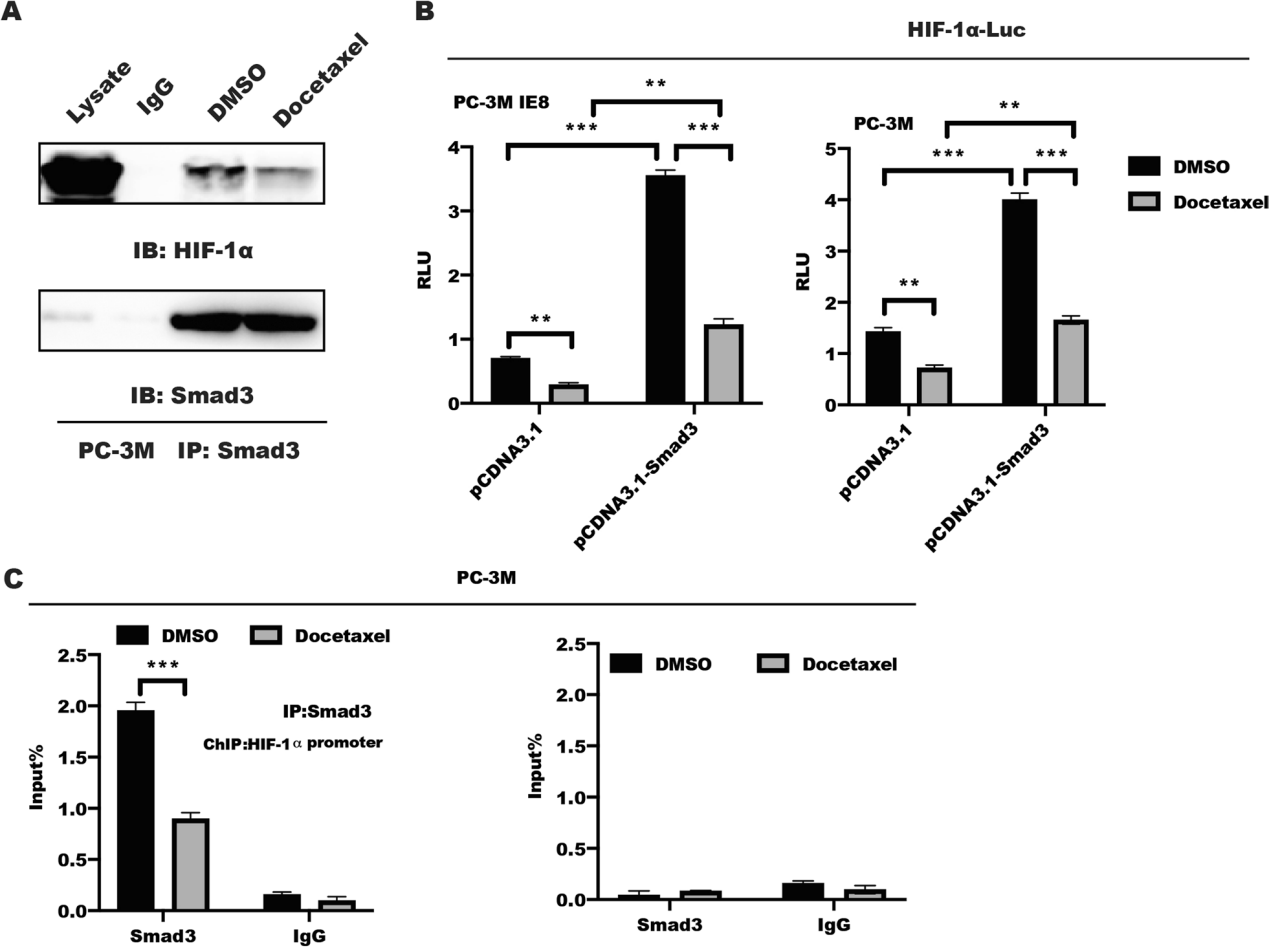
Docetaxel is crucial for binding of Smad3 to the HIF-1α promoter and subsequent HIF-1α transactivation. **A** PC-3M cells were grown to 70–80% confluence, serum starved for 24 h, then stimulated with or without 5 uM docetaxel for 1 h, whole cell lysates were immunoprecipitated (IP) with antibodies targeting endogenous Smad3 and co-precipitates with HIF-1α were detected by immunoblotting. **B** PC-3M IE8 (left panel) and PC-3M (right panel) cells were transfected with plasmid, including pCDNA3.1 and pCDNA3.1-Smad3, combined with a vector containing the HIF-1α promoter sequence driving the firefly luciferase reporter (HIF-1α -luc) in conjunction with a control Renilla luciferase expression vector. At 24 h after transfection, cells were serum starved for an additional 12 h, followed by 5 uM docetaxel treatment for 16 h. Luciferase reporter activity is presented as the fold activation relative to Renilla luciferase activity. Data represented the mean ± s.d. of three independent experiments and analyzed by two-way ANOVA with multiple comparisons, followed by Bonferroni post hoc test for significance. **P < 0.01, ***P < 0.001. **C** PC-3M cells were serum starved, and treated as indicated for 1 h, the whole cell lysates were immunoprecipitated with an anti-Smad3 antibody, co-precipitating chromosome fragments binding to Smad3 in vivo were amplified and quantified by real-time PCR. Results are presented as a ratio of the immunoprecipitated product to the input product. Left panel: real-time PCR of the Smad3-enriched HIF-1α promoter region. Right panel: real-time PCR of a nonspecific region corresponding to the exon of the HIF-1α gene enriched by Smad3 (negative control). Data represented the mean ± s.d. of three independent experiments and analyzed by two-way ANOVA with multiple comparisons, followed by Bonferroni post hoc test for significance.

The study from Xu et al. showed that Smad3 might have a central role in the transactivation of HIF-1α [11]. The above result showed that Smad3 nuclear translocation is regulated by docetaxel stimulation, leading to inhibition of direct DNA binding to target genes. Interestingly, the interaction between Smad3 and HIF-1α was disrupted in PC-3M cells in response to docetaxel stimulation (Fig. 4A). We further explored whether docetaxel-dependent expression of HIF-1α could be attributed to upregulation of Smad3 signaling. As shown in Fig. 4B, luciferase analysis showed that overexpression of Smad3 in PC-3M and PC-3M IE8 cells led to a significant upregulation of HIF-1α-specific promoter sequence (HIF-1α-luc), which was declined dramatically in PC-3M cells in response to docetaxel stimulation. Moreover, chromatin Immunoprecipitation (ChIP) followed by qPCR assay revealed that docetaxel treatment in PC-3M cells significantly decreased Smad3 binding to the promoter of HIF-1α (Fig. 4C), However, as a negative control, no binding of GAPDH, which has been shown to not bind Smad3, was observed, indicating that the IP and real-time PCR-based amplification of the HIF-1α promoter sequence was specific.

### Docetaxel/Smad3 axis suppressed tumor glycolysis and tumor growth in vivo.

**Fig. 5:**
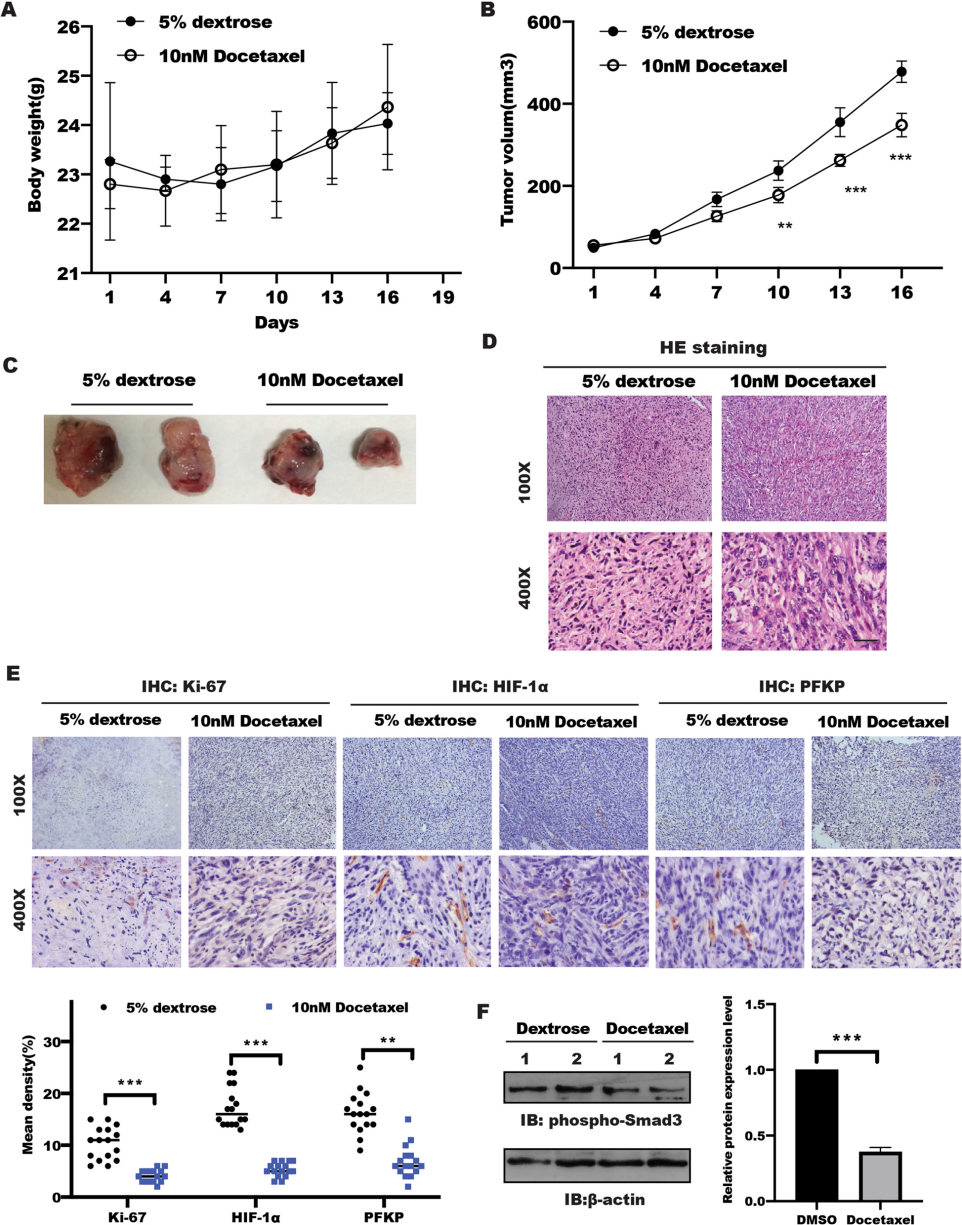
Docetaxel suppressed glycolysis and tumor growth in vivo. Null mice bearing PC-3M cell tumors were administered a daily vehicle [control group; 5% (w/v) dextrose] or Docetaxle at 10 nM for 2 weeks (4 mice per group). **A** The body weight of indicated group was measured 3 day per time, **B** tumor volume was represented at indicated time, **C** the excised tumor images were presented. **D** HE staining was used to show the tumor morphological alterations. **E** Immunohistochemistry staining for Ki-67, HIF-1α and PFKP. Magnification 400 ×, the mean density of Ki-67, HIF-1α and PFKP were quantified and analyzed by two sample t test. Data represented the mean ± s.d. **P < 0.01, ***P < 0.001. **F** Western blotting was performed to detect the phosphorylation of Smad3 (Ser213) in indicated group, β-actin served as internal control. The intensity of band was quantified, data represented the mean ± s.d. of three independent experiments and analyzed by t test, **P < 0.01, ***P < 0.001

To provide evidence into the effect of docetaxel on the tumor glycolysis and tumor growth in prostate cancer model in vivo. PC-3M cells were injected subcutaneously into nude mice as indicated group at a density of 1.0 × 10^6^ cells. Mice bearded with tumors were randomized to two groups and treated with or without docetaxel as described as shown in Fig. 5A–B. The body weight and tumor volume were recorded during the following 2 weeks. The tumor volume was significantly inhibited in docetaxel treatment group compared with that in control group (5% dextrose). Tumor size was also significantly decreased by docetaxel treatment (Fig. 5C).

Furthermore, tumor issues were processed for hematoxylin–eosin (HE) staining. Histopathological changes in the docetaxel treatment group were clearly observed (Fig. 5D). Moreover, pathologic analysis showed that cell proliferation labelled with Ki-67 was significant decreased in docetaxel-treated group compared with that in the control group by immunohistochemistry, which is attributed to decreased HIF-1α caused by docetaxel treatment (Fig. 5E). Most importantly, phosphorylation of Smad3 was reduced in docetaxel-treated group compared with that in control group (Fig. 5F). Taken together, these data provide in vivo evidence that docetaxel could inhibit prostate cancer progression through Smad3-mediated glycolysis.

## Discussion

Prostate cancer (PCa) is the second leading cause of cancer death worldwide [34], and docetaxel is the mainstay of treatment in high-volume hormone-sensitive prostate cancer and castration-resistant prostate cancer [35]. However, in this study, we have demonstrated that docetaxel repressed tumor progression through Smad3-mediated tumor glycolysis in vivo and in vitro through HIF-1α, thereby inhibiting cell proliferation. Enhanced PFKP was correlated with tumor proliferation in prostate tumor tissues compared with the normal tissues. Targeting to inhibit glycolysis could be an improvement therapy strategy in treatment of clinical prostate cancer.

Tumor glycolysis, characterized by an adaptive switch from oxidative respiration to glycolysis, even in the presence of oxygen, confers a survival advantage to cancerous cells [36, 37], which has demonstrated to be regulated by HIF-1α [20, 38, 39]. Docetaxel treatment induced redox imbalance, resulting in a specific modulation of the antioxidant response in SH-SY5Y cells, including a significant reduction of total glutathione, ascorbic acid, and an increase in both total F2-Isoprostanes and catalase activity, as compared to untreated cells [40]. What’s more, docetaxel treatment could lead to inhibit cell proliferation by targeting IGF-1 and miR-129-3p-induced-chemoresistance [24, 41]. Interestingly, In the present study we found that docetaxel treatment resulted in attenuation of HIF-1α expression, leading to decrease glycolysis and cell proliferation. Docetaxel inhibited cell proliferation by decreased tumor glycolysis due to tumor glycolysis could provide several advantages for tumor cells, such as cell proliferation, lower production of reactive oxygen species, protection from apoptosis and exertion of tumor drug resistance [42]. Interestingly, the further work revealed that, compared with vehicle, there is no significant difference of glycolysis-related gene expression at mRNA and protein level in docetaxel-resistant prostate cancer cells in response to Docetaxel treatment, which could be helpful to identify the mechanism of docetaxel-resistance in next work (Data unpublished). In addition to HIF-1α, the further work is required to explore the possible key point through which mediated glycolysis involved in docetaxel-mediated tumor glycolysis.

Smad3, a central regulator of energy reprogramming, has been reported to regulate HIF-1α expression to promote tumor glycolysis in prostate cancer cells [20], and phosphorylation of Smad3 linker domain, including Ser213, Ser204, Ser208 and Thr179, indirectly inhibits its COOH– terminal phosphorylation and subsequently suppresses tumor-suppressive pSmad3C signaling, generating resistance to the growth-inhibitory effect of TGF-β [33, 43–45]. In this study, we found that decreased Smad3 phosphorylation in PC-3M and PC-3M IE8 cells in response to docetaxel treatment, leading to inhibit Smad3 nuclear translocation and HIF-1α transactivation due to the binding of Smad3 to HIF-1α promoter was significantly reduced in PC-3M and PC-3M IE8 cell treated with docetaxel for 1 h, leading to suppress HIF-1α transactivation. Most importantly, the in vivo results showed that HIF-1α expression was also decreased in docetaxel-treated group, leading to suppress cell proliferation in animal model. These findings suggested that Smad3-HIF-1α axis plays an important role in the inhibitory effect of docetaxel in prostate cancer cells. The further work is required to address the possible mechanism of docetaxel regulated HIF-1α expression, such as ubiquitination and degradation.

## Conclusion

In summary, our study is the first to reveal the role of docetaxel-mediated tumor glycolysis in prostate cancer cell proliferation. Further studies are needed to elucidate the changes of other metabolic reprogramming, such as fatty acids metabolism, ketogenesis, polyol pathway, in docetaxel-mediated suppressive role in prostate cancer cells. However, this study highlighted the interactions between docetaxel with glucose metabolism, which may shed light on new therapeutic strategies for tumor metabolic therapy.

## Supplementary Information

**Additional file 1.** The role of docetaxel on glycolysis in prostate cancer cells.

## Data Availability

Data are original and available upon publish online.

## Abbreviations

ATP: Adenosine triphosphate
ADT: Androgen deprivation therapy
CRPC: Castration-resistant prostate cancer
CDNA: Complementary DNA
ChIP: Chromatin immunoprecipitation
CCK-8: Cell count kit-8
FBS: Fetal boive serum
GLUT1: Glucose transporter isoform 1
HK2: Hexokinase 2
HIF-1α: Hypoxia-inducible factor 1-alpha
*H. pylori*: *Helicobacter pylori*
IHC: Immunohistochemistry
LDHA: Lactate dehydrogenase A
PFKP: Phosphofructokinase, platelet
PKM2: Pyruvate kinase isozymes 2
QPCR: Quantitative polymerase chain reaction
WB: Western blotting

## References

[1] Park JH, Pyun WY, Park HW (2020). Cancer metabolism: phenotype, signaling and therapeutic targets. Cells.

[2] Xu R, Yang J, Ren B, Wang H, Yang G, Chen Y (2020). Reprogramming of amino acid metabolism in pancreatic cancer: recent advances and therapeutic strategies. Front Oncol.

[3] Fang C, Liu Y, Chen L, Luo Y, Cui Y, Zhang N (2021). alpha-Hederin inhibits the growth of lung cancer A549 cells in vitro and in vivo by decreasing SIRT6 dependent glycolysis. Pharm Biol.

[4] Almouhanna F, Blagojevic B, Can S, Ghanem A, Wolfl S (2021). Pharmacological activation of pyruvate kinase M2 reprograms glycolysis leading to TXNIP depletion and AMPK activation in breast cancer cells. Cancer Metab.

[5] Li Y, Qin J, He Z, Cui G, Zhang K, Wu B (2021). Knockdown of circPUM1 impedes cell growth, metastasis and glycolysis of papillary thyroid cancer via enhancing MAPK1 expression by serving as the sponge of miR-21-5p. Genes Genom.

[6] Tang J, Luo Y, Wu G (2020). A glycolysis-related gene expression signature in predicting recurrence of breast cancer. Aging (Albany NY).

[7] Schmidt CA, McLaughlin KL, Boykov IN, Mojalagbe R, Ranganathan A, Buddo KA (2021). Aglycemic growth enhances carbohydrate metabolism and induces sensitivity to menadione in cultured tumor-derived cells. Cancer Metab.

[8] Zhang W, Zhang X, Huang S, Chen J, Ding P, Wang Q (2020). FOXM1D potentiates PKM2-mediated tumor glycolysis and angiogenesis. Mol Oncol.

[9] Icard P, Shulman S, Farhat D, Steyaert JM, Alifano M, Lincet H (2018). How the Warburg effect supports aggressiveness and drug resistance of cancer cells?. Drug Resist Updat.

[10] Lu J, Tan M, Cai Q (2015). The Warburg effect in tumor progression: mitochondrial oxidative metabolism as an anti-metastasis mechanism. Cancer Lett.

[11] Xu W, Zeng F, Li S, Li G, Lai X, Wang QJ (2018). Crosstalk of protein kinase C epsilon with Smad2/3 promotes tumor cell proliferation in prostate cancer cells by enhancing aerobic glycolysis. Cell Mol Life Sci.

[12] Bernard K, Logsdon NJ, Benavides GA, Sanders Y, Zhang J, Darley-Usmar VM (2018). Glutaminolysis is required for transforming growth factor-beta1-induced myofibroblast differentiation and activation. J Biol Chem.

[13] Yadav H, Quijano C, Kamaraju AK, Gavrilova O, Malek R, Chen W (2011). Protection from obesity and diabetes by blockade of TGF-beta/Smad3 signaling. Cell Metab.

[14] Wang X, Xuetao X, Wu M, Wu P, Sheng Z, Liu W (2022). Inhibitory effect of roburic acid in combination with docetaxel on human prostate cancer cells. J Enzyme Inhib Med Chem.

[15] Wang D, Tang Y, Feng F, Qi M, Fang J, Zhang Y (2022). Investigation of the apoptosis-inducing effect of docetaxel by a comprehensive LC–MS based metabolomics and network pharmacology approaches. Biomed Chromatogr.

[16] Narayan RV, Subburaj K, Mahajan R (2022). Docetaxel induced hemorrhagic onycholysis. Dermatol Ther.

[17] Doktorova H, Hrabeta J, Khalil MA, Eckschlager T (2015). Hypoxia-induced chemoresistance in cancer cells: the role of not only HIF-1. Biomed Pap Med Fac Univ Palacky Olomouc Czech Repub.

[18] Moon SU, Kang MH, Sung JH, Kim JW, Lee JO, Kim YJ (2015). Effect of Smad3/4 on chemotherapeutic drug sensitivity in colorectal cancer cells. Oncol Rep.

[19] Jiang X, Tan J, Wen Y, Liu W, Wu S, Wang L (2019). MSI2-TGF-beta/TGF-beta R1/SMAD3 positive feedback regulation in glioblastoma. Cancer Chemother Pharmacol.

[20] Xu W, Zhang Z, Zou K, Cheng Y, Yang M, Chen H (2017). MiR-1 suppresses tumor cell proliferation in colorectal cancer by inhibition of Smad3-mediated tumor glycolysis. Cell Death Dis.

[21] Zhang S, Xu W, Wang H, Cao M, Li M, Zhao J (2019). Inhibition of CREB-mediated ZO-1 and activation of NF-kappaB-induced IL-6 by colonic epithelial MCT4 destroys intestinal barrier function. Cell Prolif.

[22] Li M, Zhao J, Cao M, Liu R, Chen G, Li S (2020). Mast cells-derived MiR-223 destroys intestinal barrier function by inhibition of CLDN8 expression in intestinal epithelial cells. Biol Res.

[23] Yu Y, Yang FH, Zhang WT, Guo YD, Ye L, Yao XD (2021). Mesenchymal stem cells desensitize castration-resistant prostate cancer to docetaxel chemotherapy via inducing TGF-beta1-mediated cell autophagy. Cell Biosci.

[24] Niu XB, Fu GB, Wang L, Ge X, Liu WT, Wen YY (2017). Insulin-like growth factor-I induces chemoresistence to docetaxel by inhibiting miR-143 in human prostate cancer. Oncotarget.

[25] Deng P, Li K, Gu F, Zhang T, Zhao W, Sun M (2021). LINC00242/miR-1-3p/G6PD axis regulates Warburg effect and affects gastric cancer proliferation and apoptosis. Mol Med.

[26] Zeng D, Zhou P, Jiang R, Li XP, Huang SY, Li DY (2021). Evodiamine inhibits vasculogenic mimicry in HCT116 cells by suppressing hypoxia-inducible factor 1-alpha-mediated angiogenesis. Anticancer Drugs.

[27] Dong L, Li W, Lin T, Liu B, Hong Y, Zhang X (2021). PSF functions as a repressor of hypoxia-induced angiogenesis by promoting mitochondrial function. Cell Commun Signal.

[28] Prajumwongs P, Waenphimai O, Vaeteewoottacharn K, Wongkham S, Sawanyawisuth K (2021). Reversine, a selective MPS1 inhibitor, induced autophagic cell death via diminished glucose uptake and ATP production in cholangiocarcinoma cells. PeerJ.

[29] Liu X, Liu L, Chen K, Sun L, Li W, Zhang S (2020). Huaier shows anti-cancer activities by inhibition of cell growth, migration and energy metabolism in lung cancer through PI3K/AKT/HIF-1alpha pathway. J Cell Mol Med.

[30] Kim BH, Han S, Lee H, Park CH, Chung YM, Shin K (2015). Metformin enhances the anti-adipogenic effects of atorvastatin via modulation of STAT3 and TGF-beta/Smad3 signaling. Biochem Biophys Res Commun.

[31] Yadav H, Rane SG (2012). TGF-beta/Smad3 signaling regulates brown adipocyte induction in white adipose tissue. Front Endocrinol (Lausanne).

[32] McCaskey SJ, Rondini EA, Langohr IM, Fenton JI (2012). Differential effects of energy balance on experimentally-induced colitis. World J Gastroenterol.

[33] Matsuzaki K, Kitano C, Murata M, Sekimoto G, Yoshida K, Uemura Y (2009). Smad2 and Smad3 phosphorylated at both linker and COOH-terminal regions transmit malignant TGF-beta signal in later stages of human colorectal cancer. Cancer Res.

[34] Zhu Z, Tang G, Yan J (2020). MicroRNA-122 regulates docetaxel resistance of prostate cancer cells by regulating PKM2. Exp Ther Med.

[35] Kumar S, Singh H, Das CK, Kumar R, Mittal BR (2020). Docetaxel-induced interstitial pneumonitis detected on 68Ga-PSMA PET/CT. Clin Nucl Med.

[36] Warburg O (1956). On respiratory impairment in cancer cells. Science.

[37] Koppenol WH, Bounds PL, Dang CV (2011). Otto Warburg's contributions to current concepts of cancer metabolism. Nat Rev Cancer.

[38] Yang W, Liu J, Hou L, Chen Q, Liu Y (2021). Shikonin differentially regulates glucose metabolism via PKM2 and HIF1alpha to overcome apoptosis in a refractory HCC cell line. Life Sci.

[39] Xia L, Sun J, Xie S, Chi C, Zhu Y, Pan J (2020). PRKAR2B-HIF-1alpha loop promotes aerobic glycolysis and tumour growth in prostate cancer. Cell Prolif.

[40] Micheli L, Collodel G, Moretti E, Noto D, Menchiari A, Cerretani D (2021). Redox imbalance induced by docetaxel in the neuroblastoma SH-SY5Y cells: a study of docetaxel-induced neuronal damage. Redox Rep.

[41] Zhang Y, Wang Y, Wei Y, Li M, Yu S, Ye M (2015). MiR-129-3p promotes docetaxel resistance of breast cancer cells via CP110 inhibition. Sci Rep.

[42] Liu Q, Wang L, Wang Z, Yang Y, Tian J, Liu G (2013). GRIM-19 opposes reprogramming of glioblastoma cell metabolism via HIF1alpha destabilization. Carcinogenesis.

[43] Suzuki R, Fukui T, Kishimoto M, Miyamoto S, Takahashi Y, Takeo M (2015). Smad2/3 linker phosphorylation is a possible marker of cancer stem cells and correlates with carcinogenesis in a mouse model of colitis-associated colorectal cancer. J Crohns Colitis.

[44] Kishimoto M, Fukui T, Suzuki R, Takahashi Y, Sumimoto K, Okazaki T (2015). Phosphorylation of Smad2/3 at specific linker threonine indicates slow-cycling intestinal stem-like cells before reentry to cell cycle. Dig Dis Sci.

[45] Rezaei HB, Kamato D, Ansari G, Osman N, Little PJ (2012). Cell biology of Smad2/3 linker region phosphorylation in vascular smooth muscle. Clin Exp Pharmacol Physiol.

