# Supplementary figures and images for: Docetaxel suppressed cell proliferation through Smad3/HIF-1α-mediated glycolysis in prostate cancer cells
Source: Cell Commun Signal. 2022 Dec 19;20:194. doi: 10.1186/s12964-022-00950-z (PMC9762006; doi:10.1186/s12964-022-00950-z)

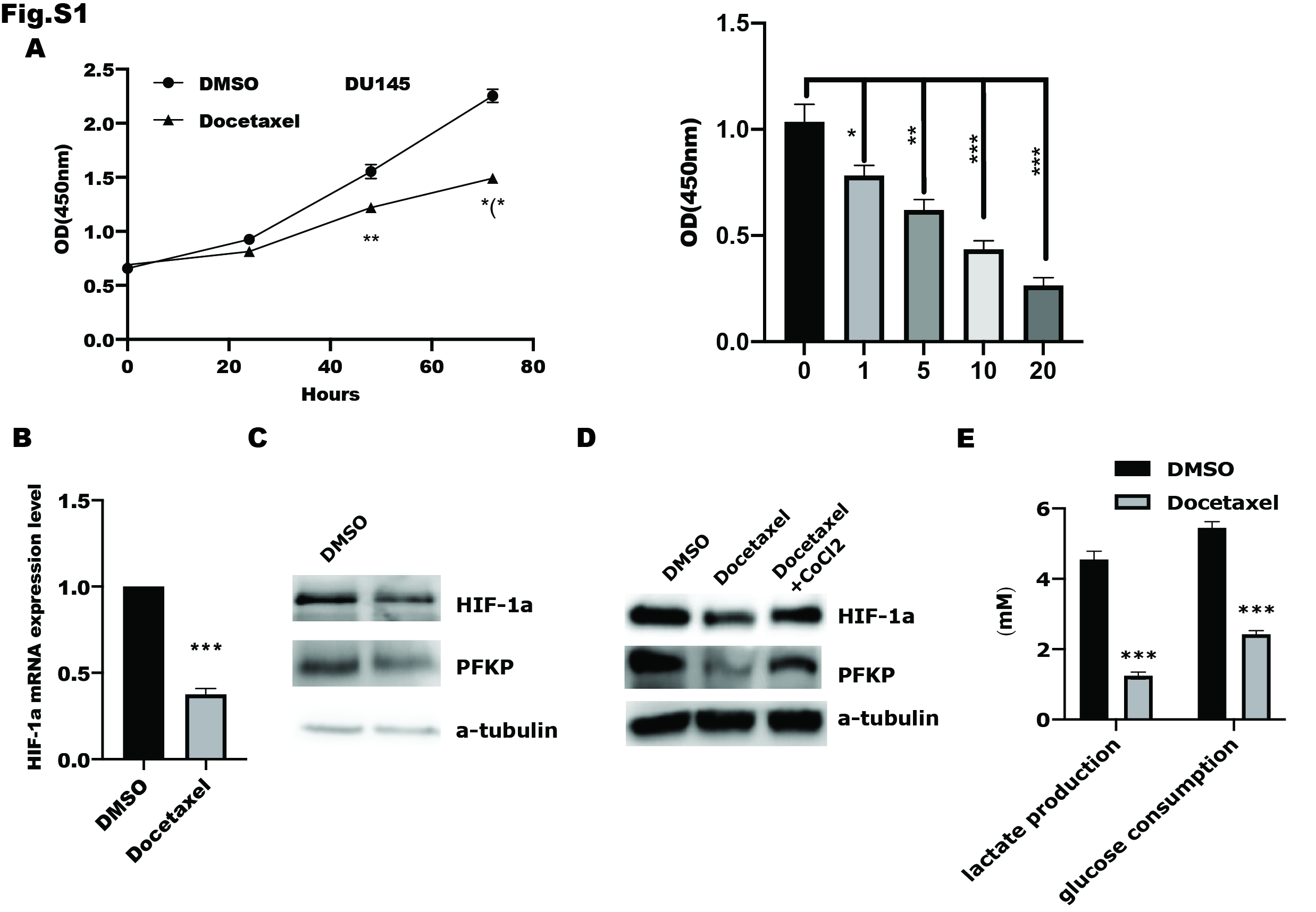

Supplement: Supplementary file 2 — Additional file 1. The role of docetaxel on glycolysis in prostate cancer cells. [file 12964_2022_950_MOESM2_ESM.jpg]
